# From Privilege to Threat: Unraveling Psychological Pathways to the Manosphere

**DOI:** 10.1007/s10508-025-03114-5

**Published:** 2025-03-21

**Authors:** Brooke Franklin-Paddock, Michael J. Platow, Michelle K. Ryan

**Affiliations:** 1 School of Medicine and Psychology, The Australian National University, Canberra, ACT 2601 Australia; 2 Global Institute for Women’s Leadership, The Australian National University, Canberra, Australia; 3 Faculty of Business and Economics, University of Groningen, Groningen, The Netherlands

**Keywords:** Online misogyny, Manosphere, Anti-feminism, Identity, Sexism, Incels

## Abstract

In this research, we explore how identity influences the adoption of misogynistic beliefs central to the manosphere, online communities known for sexism and linked to increasing extremism and real-world violence against women. Through two correlational studies (Study 1: *N* = 311; Study 2: *N* = 470), we examined how identity factors related to privilege, identification, and perceived threat from feminism predict the endorsement of manosphere attitudes. We focus on two key manosphere attitudes: anti-feminism and evolutionary beliefs about women's manipulative nature. As predicted, results showed that the less men acknowledge their privileged status relative to women, the more they feel threatened by feminists, which in turn was associated with endorsing manosphere attitudes. In Study 2, we found evidence that perceptions of status stability moderate this relationship. Men who recognized their privilege and foresee changing gender dynamics reported feeling less threatened and showed lower affinity for manosphere attitudes. We discuss the potential for mitigating the appeal of manosphere attitudes and emphasized the need for future research on conceptualizations of masculine identity and updated measures of sexism that reflect the content of contemporary gender discourse and the manosphere.

## Introduction

The denigration and harassment of women through online platforms is a prevalent and severe manifestation of sexism that has emerged with the expansion of the internet. The online communities that enable, facilitate, and promote sexist attitudes and behaviors are collectively referred to as the *manosphere* (Ging, 2019; Marwick & Caplan, 2018). Online platform algorithms, such as YouTube, expose and indoctrinate young men (and occasionally women) into manosphere ideology (Reset Australia, 2022). Concerningly, the manosphere appears to be becoming more toxic and extreme in its attitudes (Farrell et al., 2019; Horta Ribeiro et al., 2021) and has been connected to numerous mass shootings (Thorburn et al., 2022).

Existing research into the manosphere has focused on understanding the prevailing ideologies of manosphere groups (e.g., Ging, 2019; Vallerga & Zurbriggen, 2022; Van Valkenburgh, 2021). The present study extends this existing body of knowledge by examining several socialpsychological processes that may represent antecedents to specific manosphere attitudes in the general population. We apply a social identity approach to two key aspects of manosphere attitudes: anti-feminism and evolutionary beliefs about women’s manipulative nature toward men. Below, we review the literature on the manosphere, identify key psychological concepts and processes, and outline our hypotheses before presenting two quantitative online studies.^1^

### Manosphere Attitudes

Whilst acknowledging the diverse range of viewpoints within the manosphere, in the current paper we focus on two core attitudes: anti-feminism and beliefs about women’s manipulative nature toward men. Both of these revolve around the derogation of women, painting them as a real and natural threat to men, and have consistently emerged as key themes in qualitative analyses of manosphere forums (e.g., see Hopton & Langer, 2022; Vallerga & Zurbriggen, 2022). First, feminism is widely disparaged within the manosphere, portrayed as a wedge that drives men and women apart and hampers men’s economic, interpersonal, and individual prospects, among others (Gotell & Dutton, 2016; Hopton & Langer, 2022; Van Valkenburgh, 2021). For example, those in the incel community attribute their absence of sexual relationships to the rise of feminism, claiming it has reshaped society in ways that deprive men of what they are supposedly entitled to (Menzie, 2022; Whittaker et al., 2024).

Second, many within the manosphere misuse evolutionary principles to explain the motivations and behavior of men and women, as well as to justify their own misogynist views (Bachaud & Johns, 2023; Krendel, 2020; Thorburn et al., 2022; Vallerga & Zurbriggen, 2022; Van Valkenburgh, 2021). Central to this perspective is the belief that women, owing to their perceived physical vulnerabilities, are evolutionarily hardwired to be manipulative as survival mechanisms (Krendel, 2020; Sparks et al., 2024; Vallerga & Zurbriggen, 2022; Van Valkenburgh, 2021). The manosphere frequently exemplifies this view through the term hypergamy, alluding to an assumed innate tendency among women to leverage relationships with men for socioeconomic advantage, thereby undercutting men’s opportunities for success (Krendel, 2020). Although empirical research suggests that heterosexual women consider status and financial resources important partner characteristics (Buss & Schmitt, 2019; Walter et al., 2020), the extent to which this is believed to affect women’s romantic choices is significantly overemphasised in the manosphere (Costello et al., 2024).^2^

In recent years, the dissemination of manosphere attitudes has extended far beyond dedicated online forums, facilitated by the emergence of online platforms such as YouTube (Reset Australia, 2022) and TikTok (Solea & Sugiura, 2023). We are already seeing the potential consequences of this exposure, with a UK study finding 50% of school-aged boys agreed with the statement that “feminism has gone too far” (HOPE not Hate, 2020). This extended reach is concerning, given that manosphere ideology has been linked to tech-based abuse (Ging, 2019; Marwick & Caplan, 2018), real-world harassment (Thorburn et al., 2022) and acts of violent extremism (Latimore & Coyne, 2023). Hence, with the increasingly widespread dissemination and endorsement of manosphere attitudes, it is important for research to understand what factors are associated with an increased likelihood of acceptance.

### The Role of Identities in Manosphere Attitudes

Manosphere attitudes are deeply rooted in the intergroup context of men relative to (and often “versus”) women. Unlike women, men are seen as inherently rational and in control (Ging, 2019; Vallerga & Zurbriggen, 2022; Van Valkenburgh, 2021). Core to this is the sense of a positive group-based social identity, that of being a man, which offers value, meaning, and distinction from women (Van Valkenburgh, 2021). The importance of this social identity is typified by discussions on how to become an alpha male (i.e., the idealised subgroup identity) that fill the manosphere—such as *the Rational Male* blog or Reddit forums for *How to Manage Your Bitches.* While certain groups, notably incels (i.e., involuntary celibates), feel hopeless in their ability to achieve alpha male status, there is a consensus that, irrespective of their position in the male hierarchy, men remain inherently superior to women (Dickel & Evolvi, 2022). Thus, being a man remains a central part of individuals’ self-concepts in the manosphere. Crucially, it is an identity often perceived and experienced as under threat from feminism (Dickel & Evolvi, 2022; Krendel, 2020).

Given the significant role that masculine identities play in the manosphere and the perception that feminism is a threat to men’s identity, utilising a social identity threat framework (e.g., Branscombe et al., 1999; Tajfel & Turner, 1986) offers a unique and appropriate lens to investigate the psychology underpinning manosphere attitudes. Building on existing theory and research, the subsequent sections explore how feminism, through its perceived challenge to men’s status privileges over women, may be interpreted and subjectively experienced by some men as a threat to their male social identities. Moreover, we outline how these social identity threats may form key pathways to manosphere attitude endorsement in the general population.

#### Perceptions of Men’s Privilege

The concept of victimhood is central to identities within the manosphere (Krendel, 2020). While objective data consistently indicates that women as a group face systemic disadvantages compared to men—such as lower average pay (Workplace Gender Equality Agency, 2022), restricted bodily autonomy (Reingold & Gostin, 2016), and higher rates of partner violence, economic abuse, and emotional abuse (Australian Bureau of Statistics, 2022)—manosphere attitudes include the belief that society is gynocentric, meaning it favours women over men (Krendel, 2020; Mingo & Fernández, 2023). It is crucial to note that men's broader societal privileges on average do not diminish the intersectional disadvantages faced by some men (e.g., related to race or socioeconomic status; for review of intersectional disadvantage, see Loets, 2024). Nor do these privileges invalidate the challenges men experience in their collective identity, particularly as gender roles have evolved in society (for review, see Kimmel, 2018). Further, there is a growing body of literature on incels that highlights a number of sociocultural, relational, and mental health challenges (e.g., loneliness) faced by these men (Brooks et al., 2022; Costello et al., 2022, Costello & Thomas, 2025; Sparks et al., 2024). However, by recasting men as the primary victims, the manosphere seeks to shift societal discourse, suggesting that men, as a group, are more disadvantaged than, and persecuted by, women.

This narrative shift seems to have found traction in the general population, with a growing portrayal of men as underprivileged and discriminated against in society (Carian, 2022; Hodson et al., 2022; Zehnter et al., 2021)—a trend that is growing among school-aged boys (HOPE not Hate, 2020). Concerningly, reluctance to acknowledge privilege has been empirically associated with the expression of hostile sexism (overtly anti-women, e.g., demonizing the motivations of women; Case, 2007; Glick & Fiske, 1996), underscoring the societal implications of privilege denial.

Building on this, insights from the literature on social identity threat serve as a valuable framework for understanding why men who perceive themselves as having less privilege than women may opt to derogate women (e.g., Branscombe et al., 1999; Ellemers et al., 2002; Hunter et al., 1997; Scheepers, 2009). Central to social identity theory is the notion that people derive an important part of their identity from the groups they belong to (social identity; Tajfel & Turner, 1986). Hence, individuals identifying as part of a (perceived) underprivileged, low-status group experience threats to their identity (Branscombe et al., 1999). In response, they may derogate perceived oppressors to reaffirm and bolster their positive identity (Branscombe et al., 1999; Platow et al., 1997; Scheepers, 2009). Thus, while the manosphere may deploy derogatory representations of women as a tactical measure against feminism, men in the broader community (i.e., those not necessarily part of the manosphere community) who do not acknowledge their privileged status relative to women could find resonance with manosphere attitudes precisely as a means to reinforce their identity as a man. Thus, in our first hypothesis, we predict that the less men acknowledge their privilege relative to women, the more likely they are to endorse manosphere attitudes (H1; Table 1).

**Table 1:** Hypothesized relationships between privilege acknowledgement, status stability, threat, and endorsement of manosphere attitudes

Hypotheses	
**Direct effects on manosphere attitudes**	
H1	The less men acknowledge their privilege relative to women, the more likely they are to endorse manosphere attitudes
H2	There will be a disordinal interaction between men's acknowledgement of their privilege and the perceived status stability between men and women. When men have higher levels of privilege acknowledgement, we expect that endorsement of manosphere attitudes will be higher when the status relationship between men and women is perceived as unstable compared to when it is perceived as stable. Conversely, when men have lower levels of privilege acknowledgement, we anticipate that endorsement of manosphere attitudes will be higher when the status relationship between men and women is perceived as stable compared to when it is perceived as unstable
H3	The relationship between privilege acknowledgement, status stability, and manosphere attitudes (as described in Hs 1 and 2) will be observed primarily, if not solely, in men with relatively high social identification as a man compared to men with relatively low social identification
**Experience of threat as mediator**	
H4a	Lower privilege acknowledgement by men will be associated with increased feelings of threat from feminism, which will, in turn, predict greater endorsement of manosphere attitudes
H4b	The relationship between lower levels of privilege acknowledgement and threat (H4a) will be moderated by perceptions of intergroup status stability. The nature of this interaction is expected to align with the predictions made in H2: when men have higher levels of privilege acknowledgement, we expect that threat will be higher when the status relationship between men and women is perceived as unstable compared to when it is perceived as stable. Conversely, when men have lower levels of privilege acknowledgement, we anticipate that threat will be higher when the status relationship between men and women is perceived as stable compared to when it is perceived as unstable
H4c	The relationship between privilege acknowledgement, status stability, and threat (H4b) will be observed primarily, if not solely, in men with relatively high male social identification compared to men with relatively low male social identification

#### The Stability of the Status Relations between Men and Women

In the manosphere, a lack of male privilege acknowledgement often intersects with the belief that men must awaken to and accept the reality of men’s oppression by women (Ging, 2019; Van Valkenburgh, 2021). Although widespread adherence to the extremity of this belief is unlikely, it is conceivable that a substantial number of men may have been exposed to, and agree with, the growing belief that feminism has progressed to the point where women are now more privileged than men (Carian, 2022; Zehnter et al., 2021). In the context of our research, centred on social identity threat, we view a key aspect of this belief as reflecting perceived stability in the relational status of men and women. Building on past research on social identity threats in lower-status groups (e.g., Scheepers, 2009), we posit that the perceived stability of men’s lower-status relative to women threatens the positive distinctiveness of men’s identities. Hence, when perceived status stability is combined with a lack of acknowledgement of privilege, we hypothesise that this will represent a key psychological pathway to the endorsement of manosphere attitudes in the general population.

However, for men who acknowledge their privileged status, socialpsychological theories (Stephan & Stephan, 2000; Tajfel & Turner, 1986) suggest another pathway: the role of status instability (i.e., perception that status hierarchies are changing). Research in this realm has shown that when members of high-status groups discern their status as unstable, it can spur heightened prejudice toward groups seen as driving this instability (e.g., Bettencourt et al., 2001; Cunningham & Platow, 2007; Rivera-Rodriguez et al., 2022). A study by Morton et al. (2009) exemplified this, finding that for highly sexist men, making salient the rising status of women significantly increased their endorsement of essentialist gender differences compared to control (status stable condition). Together with the above, this highlights that experiencing identity threat may come from two combinations of relative privilege acknowledgement and status stability. We outline the predicted nature of this interaction (H2) in Table 1.

#### Feminism as a Threat to Men’s Social Identity

As we indicated in our review above, manosphere ideology includes the perception that feminism is an attack on men’s social identity. However, it is important to note that there is individual variability within men in the extent to which their gender defines their self-concept (i.e., their social identification). Research examining the role of social identification (e.g., Bosson & Michniewicz, 2013; Doosje & Ellemers, 1997; Maass et al., 2003; Rivera-Rodriguez et al., 2022) supports Branscombe et al.’s (1999) taxonomy of social identity threats, which proposes that it is only high-identifying individuals who interpret challenges to the status quo as a threat to an essential part of their identity. This threat can evoke backlash, in the form of adopting attitudes that devalue groups posing a threat to the status quo (Branscombe et al., 1999). In comparison, group members whose identity is less defined by their group membership (i.e., low identifiers) are less likely to feel threatened and display backlash (Branscombe et al., 1999). For instance, a study by Maass et al., (2003) found that, when exposed to a description of a feminist woman, high-identifying men were more likely to sexually harass her (e.g., sending explicit imagery), while low-identifying men did not exhibit such behavior. Applying this to the current research, we predict the relationships described in H1 and H2 will be stronger among men with higher male social identification compared to those with lower male social identification (H3; Table 1).

#### Experience of Threat

**Fig. 1:**
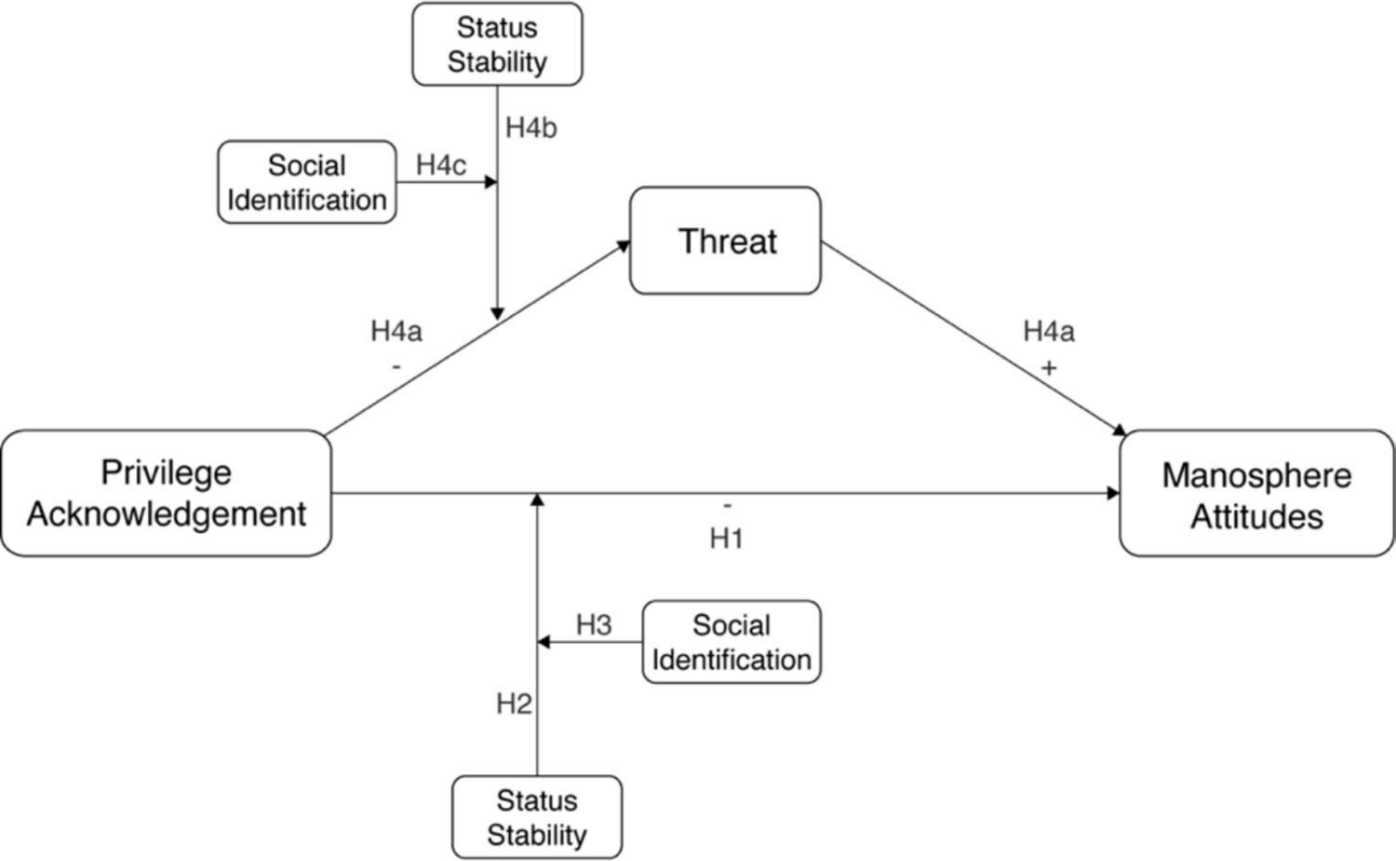
Hypothesized relationships between privilege acknowledgement, status stability, social identification, threat, and manosphere attitudes. *Note* for visual simplicity, the figure depicts manosphere attitudes toward feminism and evolution as one

Having discussed how threats to men's identity may arise from perceptions of their privileged status relative to women, we must also consider how men experience this threat. Given the vilification of feminism and feminists within the manosphere (Marwick & Caplan, 2018), we suggest the pathway from privilege denial to manosphere misogyny may be explained, at least in part, by a heightened experience of threat from feminism. Indeed, empirical evidence has consistently supported the mediating role the subjective experience of threat has on increasing prejudice in a variety of contexts (Stephan, 2014). Accordingly, we predict the relationship between privilege acknowledgement, status stability, and manosphere attitudes (H1–3) will be mediated by the experience of threat from feminism (H4a-c; Table 1). We visualise our hypotheses in the path model displayed in Fig. 1.

### The Present Research

In the present research, we explored our hypotheses through two correlational studies in the broader population. We intentionally did not sample specifically from those in or aligned with manosphere beliefs. Had we taken this latter approach, our work would have effectively been tautological (i.e., showing that manosphere men express manosphere beliefs). In contrast, not only does our broader sample avoid this tautology directly, but the nature of our design inherently recognizes the diversity among men and their attitudes and beliefs. Our hypotheses articulate where and when we anticipate at least some of this diversity. The first study served as an initial examination of our hypotheses, while we preregistered the second study and aimed to replicate the first study's findings with a larger sample size.^3^ We limited participation to those residing in the USA, given the relevance of this demographic to manosphere activity (Horta Ribeiro et al., 2021). Below, we present the methods and results of both studies.

## Method

### Participants

We recruited samples of US American self-identifying men (Study 1, *N* = 330; Study 2, *N* = 500) using Amazon Mechanical Turk (MTurk) via third-party Cloud Research. Crowdsourced sampling using MTurk has become widely used in academic research over the past decade (Agley et al., 2022). One of the main benefits of MTurk is that, compared to sampling university students, it offers a more diverse and representative population of participants (Burnham et al., 2018). Having said this, recent research has highlighted potential data quality issues relating to inattentive, dishonest, and potential bot responses (Chmielewski & Kucker, 2020). Hence, to ensure high-quality data, we first screened the responses to identify non-human responses. Following Qualtrics recommendations for using embedded fraud-detection data, we removed suspected bots (Study 1, *n* = 4; Study 2, *n* = 4). Although we utilised screening criteria only to sample men, participants who reported being a gender other than a man were identified and removed (Study 1, *n* = 6; Study 2, *n* = 12). Considering the risk of careless responding in online environments, we deemed responses completed in less than 3 or greater than 50 min (Study 1, *n* = 4; Study 2, *n* = 6) to be unreasonable and removed them (Meade & Craig, 2012), and we removed participants who failed at least two out of five attention checks (Study 1, *n* = 5; Study 2, *n* = 7) (Marjanovic et al., 2014). In Study 2, we removed one participant for missing data, having responded to only one of six anti-feminist items. The final samples of men (*N* = 311 for Study 1, *N* = 470 for Study 2) were predominantly heterosexual (Study 1 90%, Study 2 90%) and had an age range of 20–83 years (*M* = 43.6, *SD* = 13.9) in Study 1, and 19–79 years (*M* = 40.2, *SD* = 12.2) in Study 2. We present additional demographic data in Online Resource 1, available at https://osf.io/2wek6/?view_only=0a83235d35db48eab9e29e555470a262.

### Measures and Procedure

Before data collection, we gained approval from the Australian National University’s research ethics committee (Protocol 2022/159). To begin, participants responded to an advertisement on MTurk entitled “Attitudes toward Yourself and Society”. We directed eligible participants to the Qualtrics survey, which began by asking them to read and provide informed consent. Participants then responded to the following measures using a 7-point Likert scale (1 = *strongly disagree* to *7* = *strongly agree*).^4^

**Social Identification** We administered Mael and Ashforth’s (1992) six-item Social Identification Scale to measure participants’ social identification as a man. We adapted items to refer specifically to social identification as a man (e.g., “When someone criticizes men it feels like a personal insult”) and averaged them to create a measure of each individual’s level of social identification (Study 1, α = 0.86; Study 2, α = 0.88). Higher scores indicate higher levels of social identification.

**Privilege Acknowledgement** We used a seven-item Male Privilege Awareness Scale (Case, 2007) to measure participants’ perception of the relative gender status privileges between men and women. Examples of items in this scale include: “Men are at an advantage because they hold most of the positions of power in society” and “Women are advantaged and men are currently at a disadvantage” (reverse scored). After reverse coding relevant items, we created a composite variable by averaging the items (Study 1, α = 0.91; Study 2, α = 0.89), with higher scores indicating acknowledgement of male privilege and lower values indicating denial of male privilege.

**Status Stability** We measured the perceived stability of intergroup status using four items developed by Morton et al. (2009). We found that only two of these items (“I cannot imagine the relationship between men and women being any different” and “the relationship between men and women is likely to change” reverse scored) loaded onto a single factor in an exploratory factor analysis (loadings > 0.41). We averaged these two items to form a composite measure of status stability (Study 1, *r* = 0.42, *p* < 0.001; Study 2, *r* = 0.31, *p* < 0.001), given the theoretical relevance of the construct. Higher values of status stability indicate higher perceived stability in the relationships between men and women.

**Experience of Threat** To measure the level of threat participants experienced in response to feminism, we designed a bespoke measure adapted from other similar measures (e.g., Marteau & Bekker, 1992; Watson et al., 1988) that asked participants to respond to the statement: “When I interact with feminists, I feel…”. Underneath this statement, we presented eight anxiety-related emotions (“…worried”, “…anxious”, “…threatened”, “distressed”, “…angry”, “…scared”, “…stressed”, “…indifferent”) and five threat-antithetical emotions to reduce response bias (“…hopeful”, “…empowered”, “…informed”, “…safe”, “…calm”). After reverse coding the threat-antithetical emotions, we averaged items to create a threat score (Study 1, *α* = 0.89; Study 2, *α* = 0.87) so that higher values indicate a higher experience of threat.

**Manosphere Attitudes **We asked participants to respond to two manosphere statements; each was created for this study and designed to capture the key attitudes towards evolution and feminism expressed within manosphere forums (based upon the work of Krendel, 2020; Van Valkenburgh, 2021). These were “Evolution has programmed women to continually test men’s…” and “Feminism has created a barrier between men and women that inhibits men’s….”. We presented six items under each statement: (1) “…abilities”, (2) “…success”, (3) “…achievement”, (4) “…rational thinking”, (5*)* “…strength”, and (6) “…abilities to start a family.” Hence, each manosphere attitude was comprised of six items. We did not average these items, as manosphere attitudes were estimated using CFA with the planned SEM (see Results).

**Demographic Questions** Finally, we asked participants demographic questions (e.g., age, education, relationship status), presented a debriefing page explaining the study’s aims, and provided a random completion code for payment.

## Results

We report the results of Study 1 and Study 2 together, as identical analyses were applied to both datasets. Study 1 was primarily a scoping study; as such, the sample size in Study 1 was not predetermined to test specific hypotheses but was expected to provide enough data to observe potential relationships. A post-hoc power analysis using G*Power (version 3.1) showed that, with a sample size of *N* = 311 and an alpha level of 0.05, Study 1 had 80% power to detect small effect sizes (*f*^2^ ≥ 0.026). Study 2 aimed to replicate the findings of Study 1 by testing the same hypotheses on a newly collected sample. Although our preregistered target was a minimum sample size of 500, financial and time constraints resulted in a final sample size of *N* = 470. To assess the implications of this sample size reduction, we conducted a post-hoc power analysis using G*Power. This analysis indicated that, with the achieved sample size of *N* = 470 and an alpha level of 0.05, Study 2 retained 80% power to detect small effect sizes (*f*^2^ ≥ 0.017).

### Planned Data Analysis

We completed data analysis using JASP 0.17.2.1. We used an SEM with Lavaan R code (Rosseel, 2012) to test our hypothesised direct and indirect effects (summarized in Table 1) and confirm the factor structure of manosphere attitudes. Due to violations of multivariate normality (Mardia’s normalised coefficient *p* < 0.001), we estimated the measurement model using maximum likelihood and tested using Satorra–Bentler scaled chi-square (Satorra & Bentler, 1994), as recommended by Tabachnick and Fidell (2014). We inferred relatively good model fit from CFI values ≥ 0.95 and RMSEA values close to 0.06 (Hu & Bentler, 1999). We then investigated interactions identified in the path model at higher and lower values (± 1 *SD* from the mean) of the predictor variables using simple slope analysis to identify what drove the interaction (Dawson, 2014). Table 2 presents means, standard deviations, and bivariate correlations.

**Table 2:** Descriptive statistics and bivariate correlations in Studies 1 (*N* = 311) and 2 (*N* = 470)

Variable	*M*	*SD*	1	2	3	4	5
Study 1							
1. Privilege Acknowledgement	4.38	1.36					
2. Status Stability	3.32	1.27	− .44***				
3. Social Identification	3.36	1.27	− .14*	.18*			
4. Threat	3.35	1.09	− .42***	.24***	.29***		
5. MAE	3.92	1.65	− .26***	.16**	.46***	.44***	
6. MAF	3.24	1.68	− .40***	.25***	.52***	.56***	.63***
Study 2							
1. Privilege Acknowledgement	4.23	1.33					
2. Status Stability	3.32	1.22	− .23***				
3. Social Identification	3.63	1.33	− .13**	.12*			
4. Threat	3.55	1.06	− .35***	.16***	.23***		
5. MAE	3.94	1.78	− .26***	.13**	.46***	.33***	
6. MAF	3.43	1.73	− .37***	.18***	.49***	.49***	.61***

MAE = manosphere attitudes toward evolution. MAF = manosphere attitudes toward feminism. For the purpose of descriptive statistics, we averaged the individual responses from each participant to the MAF items (Study 1, α = .97; Study 2, α = .97) and MAE items (Study 1, α = .96; Study 2, α = .96) to construct two composite variables, wherein higher scores indicate greater endorsement of manosphere attitudes. All scales have a midpoint of 4

^*^ *p* < .05, **p < .01, ***p < .001

### Structural Equation Model

Prior to analysis, we centered all predictor variables to control for multicollinearity.

#### Model Estimation

The hypothesised model fitted the data relatively well in the preliminary examination Study 1 (Satorra-Bentler χ^2^(133, N = 311) = 190.66, *p* < 0.001, CFI = 0.97, RMSEA = 0.06, 90% CI [0.05, 0.07]) and was replicated in the preregistered Study 2 (Satorra-Bentler χ^2^(123, N = 470) = 262.18, *p* < 0.001, CFI = 0.96, RMSEA = 0.07, 90% CI [0.06, 0.08]). We found support for the hypothesised two-factor structure of manosphere attitudes (standardised factor loadings > 0.82, *p*s < 0.001). Between-factor covariance was significant (Study 1, standardised covariance = 0.42, *p* < 0.001; Study 2, standardised covariance = 0.44, *p* < 0.001). However, an alternative one-factor model did not fit the data well (Study 1, Satorra–Bentler χ^2^(142, N = 311) = 1798.33, *p* < 0.001, CFI = 0.68, RMSEA = 0.19; Study 2, Satorra–Bentler χ^2^(131, N = 470) = 2519.83, *p* < 0.001, CFI = 0.68, RMSEA = 0.20) and explained less variance (Study 1, R^2^ = 0.53; Study 2, R^2^ = 0.36) compared to the two-factor model (Study 1, R^2^_anti-fem_ = 0.51, R^2^_evol_ = 0.33; Study 2, R^2^_evol_ = 0.47, R^2^_anti-fem_ = 0.29). Thus, we retained the hypothesized model in both studies, and we examined direct and indirect effects of predictor variables (below).

#### Direct Effects on Manosphere Attitudes

With the structural model, we tested the main effects and interactions of privilege acknowledgement, status stability, and social identification on threat and manosphere attitudes towards feminism and evolution. We summarize the results in Table 3. Offering support for H1, inspection of individual parameters demonstrated that lower acknowledgement of men’s privileged status relative to women was associated with higher endorsement of manosphere attitudes towards feminism in both Study 1 and Study 2 (Study 1, *p* < 0.001; Study 2, *p* < 0.001). We found a similar trend for the effect of privilege acknowledgement on manosphere attitudes towards evolution in the preliminary Study 1 (*p* = 0.304), which became statistically significant in Study 2 (*p* = 0.001). Social identification and threat were also significant positive predictors of manosphere attitudes towards feminism and evolution across both studies (Study 1, *p*s < 0.001; Study 2, *ps* < 0.001).

**Table 3:** Summary of direct effects to test hypotheses in Study 1 (*N* = 311) and Study 2 (*N* = 470)

			Study 1		Study 2		Test
			Estimate	95% CI	Estimate	95% CI	
PA	→	Anti-fem	− 0.22***	[− 0.34, − 0.09]	− 0.26***	[− 0.35, − 0.16]	H1
	→	Evolution	− 0.08	[− 0.22, 0.07]	− 0.19**	[− 0.31, − 0.08]	H1
	→	Threat	− 0.29***	[− 0.38, − 0.20]	− 0.23***	[− 0.30, − 0.16]	H4a
SS	→	Anti-fem	0.09	[− 0.04, 0.22]	0.09	[− 0.01, 0.19]	
	→	Evolution	0.0004	[− 0.15, 0.15]	0.04	[− 0.08, 0.16]	
	→	Threat	0.02	[− 0.08, 0.12]	0.06	[− 0.02, 0.13]	
SID	→	Anti-fem	0.49***	[0.37, 0.60]	0.51***	[0.42, 0.60]	
	→	Evolution	0.46***	[0.32, 0.60]	0.54***	[0.42, 0.65]	
	→	Threat	0.22***	[0.13, 0.30]	0.14***	[0.07, 0.21]	
Threat	→	Anti-fem	0.56***	[0.42, 0.71]	0.48***	[0.36, 0.60]	H4a/b/c
	→	Evolution	0.48***	[0.31, 0.65]	0.32***	[0.17, 0.47]	H4a/b/c
PA*SS	→	Anti-fem	0.14***	[0.06, 0.22]	0.10**	[0.03, 0.16]	H2
	→	Evolution	0.07	[− 0.02, 0.17]	0.04	[− 0.04, 0.13]	H2
	→	Threat	− 0.002	[− 0.07, 0.06]	0.08**	[0.03, 0.13]	H4b
SS*SID	→	Anti-fem	0.09	[− 0.005, 0.18]	0.03	[− 0.4, 0.10]	
	→	Evolution	0.02	[− 0.9, 0.13]	− 0.006	[− 0.09, 0.08]	
	→	Threat	− 0.09*	[− 0.16, − 0.02	0.001	[− 0.04, 0.06]	
PA*SS*SID	→	Anti-fem	0.06*	[0.01, 0.11]	0.08***	[0.03, 0.1s3]	H3
	→	Evolution	0.002	[− 0.06, 0.06]	0.01	[− 0.06, 0.07]	H3
	→	Threat	0.03	[− 0.01, 0.07]	0.02	[− 0.02, 0.06]	H4c

Estimates are presented as unstandardised regression coefficients. PA = male privilege acknowledgement. SS = status stability. SID = social identification. Threat = experience of threat when interacting with feminists. Evolution = manosphere attitudes towards evolution (latent variable). Anti-fem = manosphere attitudes towards feminism (latent variable)

^*^ *p* < .05, **p < .01, ***p < .001

Alone, perceived status stability did not significantly predict manosphere attitudes towards feminism or evolution in Study 1 or Study 2. However, in Study 1 and Study 2, we found that perceived status stability moderated the effect for privilege acknowledgement, such that a significant two-way interaction between privilege acknowledgement and perceived status stability predicted manosphere attitudes towards feminism (Study 1, *p* < 0.001; Study 2, *p* = 0.004). This effect was further moderated by a significant three-way interaction, observed in both Study 1 and Study 2, between privilege acknowledgement, perceived status stability, and social identification that predicted manosphere attitudes towards feminism (Study 1, *p* = 0.017; Study 2, *p* < 0.001).

**Fig. 2:**
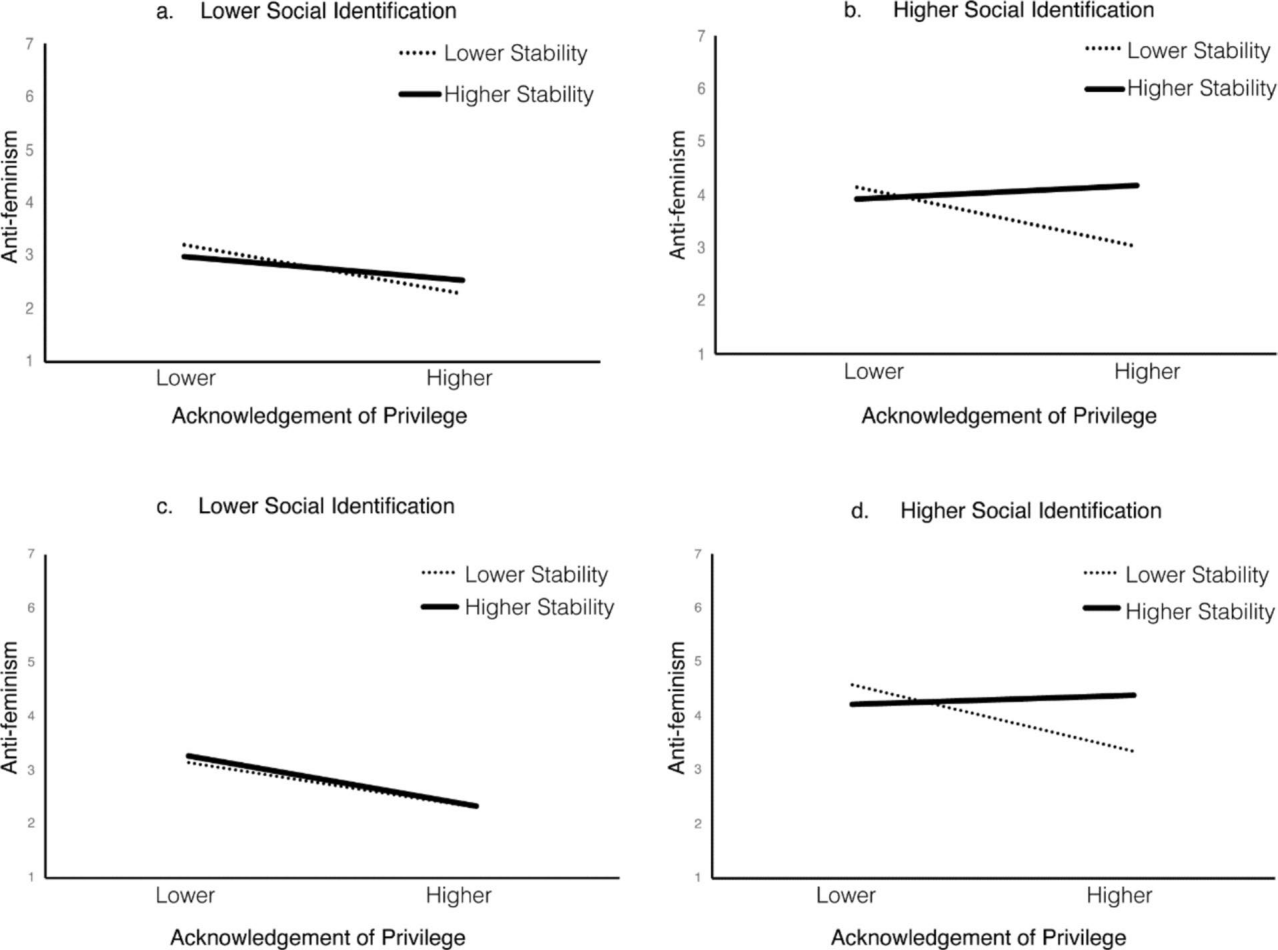
Relationship between privilege acknowledgement, status stability, and anti-feminism as qualified by **a** Lower social identification and **b** Higher social identification in Study 1 and **c** Lower social identification and **d** Higher social identification in Study 2

We used a follow-up simple slope analysis to investigate whether the effect of privilege acknowledgement on manosphere attitudes towards feminism was negative under perceived status stability (+ 1 *SD*) and positive under perceived status instability (-1 *SD*), as predicted in H2. Furthermore, we explored whether this effect was only evident in highly identifying men, as predicted in H3. We present the results in Fig. 2.

**Fig. 3:**
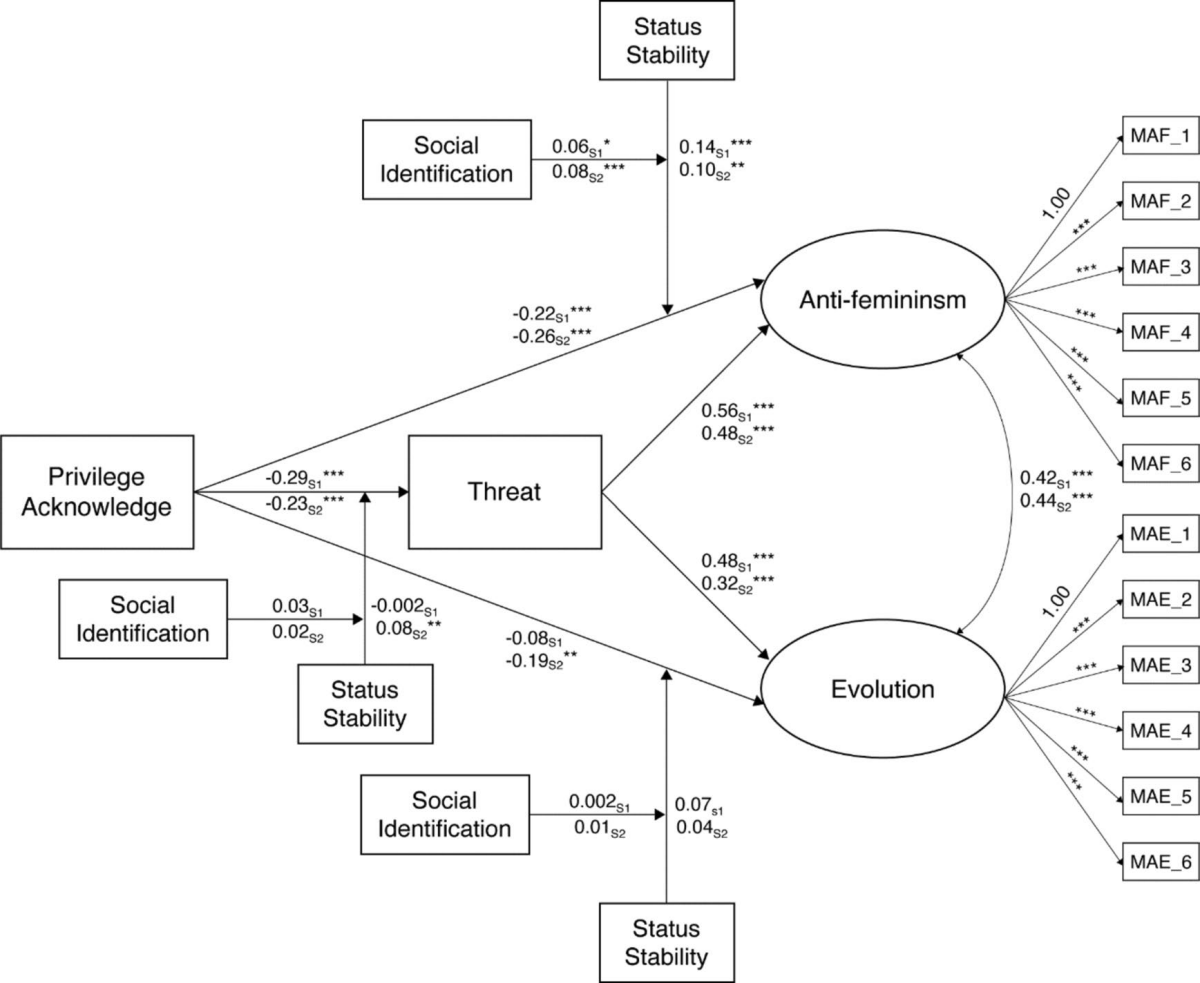
Identity threat pathways to manosphere attitudes: Structural equation model results for Study 1 and Study 2. *Note* for visual clarity, this figure does not present the main effects of status stability and social identification. Please refer to Table 3 for these results. _S1_ = results of Study 1, _S2_ = results of Study 2. *p < .05, **p < .01, ***p < .001

Offering partial support for H3, we found no interaction between privilege acknowledgement and perceived status stability in lower-identifying men in either study (Study 1, *B*_*SlopeDif*_ = 0.17, *p* = 0.072; Study 2, *B*_*SlopeDif*_ = -0.03, *p* = 0.803). In contrast, the interaction between privilege acknowledgement and perceived status stability was significant among highly identifying men in both studies (Study 1, *B*_*SlopeDif*_ = 0.50, *p* = 0.001; Study 2, *B*_*SlopeDif*_ = 0.52, *p* < 0.001). As shown in Fig. 2, a similar interaction pattern was obtained in the preliminary Study 1 and the preregistered Study 2. This interaction was primarily driven by the different impact that perceptions of status stability (versus instability) had on highly identifying men’s anti-feminist attitudes (Fig. 2b, d). However, the role of status stability did not offer support for H2. The relationship between privilege acknowledgement and anti-feminism was non-significant among highly-identified men who reported intergroup status as stable (Study 1, *B* = 0.09, *p* = 0.459; Study 2, *B* = -0.06, *p* = 0.538). Moreover, contrary to H2, for men with higher levels of social identification the relationship between privilege acknowledgement and anti-feminism exhibited a strong and negative effect under perceived status instability (Study 1, *B* = − 0.41, *p* < 0.001; Study 2, *B* = − 0.47, *p* < 0.001). Among highly identifying men who perceived the status quo as relatively unstable, the more they acknowledged their privilege, the less likely they were to endorse manosphere attitudes towards feminism.

#### The Mediating Role of Threat on Manosphere Attitudes

To examine Hypothesis 4a, we calculated the indirect effects of privilege acknowledgement on manosphere attitudes through threat. We summarize the results in Table 4 and present the structural model in Fig. 3. In support of H4a, threat partially mediated the relationship between privilege acknowledgement and manosphere attitudes (indirect effect Study 1, *p* < 0.001; Study 2, *p* < 0.001). Bivariate correlations between privilege acknowledgement and manosphere attitudes (Table 2) reduced in magnitude when accounting for the effect of perceived threat. Hence, lower levels of privilege acknowledgement predicted a higher experience of threat when interacting with feminists, which in turn predicted higher manosphere attitude endorsement.

**Table 4:** Summary of indirect effects for Study 1 (*N* = 311) and Study 2 (*N* = 470)

					Study 1		Study 2		Test
					Estimate	95% CI	Estimate	95% CI	
Privilege	→	Threat	→	Anti-fem	− 0.16***	[− 0.25, − 0.10]	− 0.11***	[− 0.17, − 0.07]	H4a
			→	Evolution	− 0.14***	[− 0.22, − 0.08]	− 0.07***	[− 0.12, − 0.04]	
PA*SS	→	Threat	→	Anti-fem	− 0.001	[− 0.04, 0.04]	0.04**	[0.01, 0.07]	H4b
			→	Evolution	− 0.001	[− 0.04, 0.03]	0.02*	[0.01, 0.05]	
PA*SS*SID	→	Threat	→	Anti-fem	0.02	[− 0.01, 0.05]	0.01	[− 0.01, 0.03]	H4c
			→	Evolution	0.01	[− 0.01, 0.04]	0.01	[− 0.01, 0.02]	

Privilege/PA = male privilege acknowledgement. *SS* status stability, *SID* social identification, Threat = experience of threat when interacting with feminists. Test = hypothesis tested via indirect effect. 95% CI = 5000 95% bootstrapped confidence intervals

^*^p < .05, **p < .01, ***p < .001

**Fig. 4:**
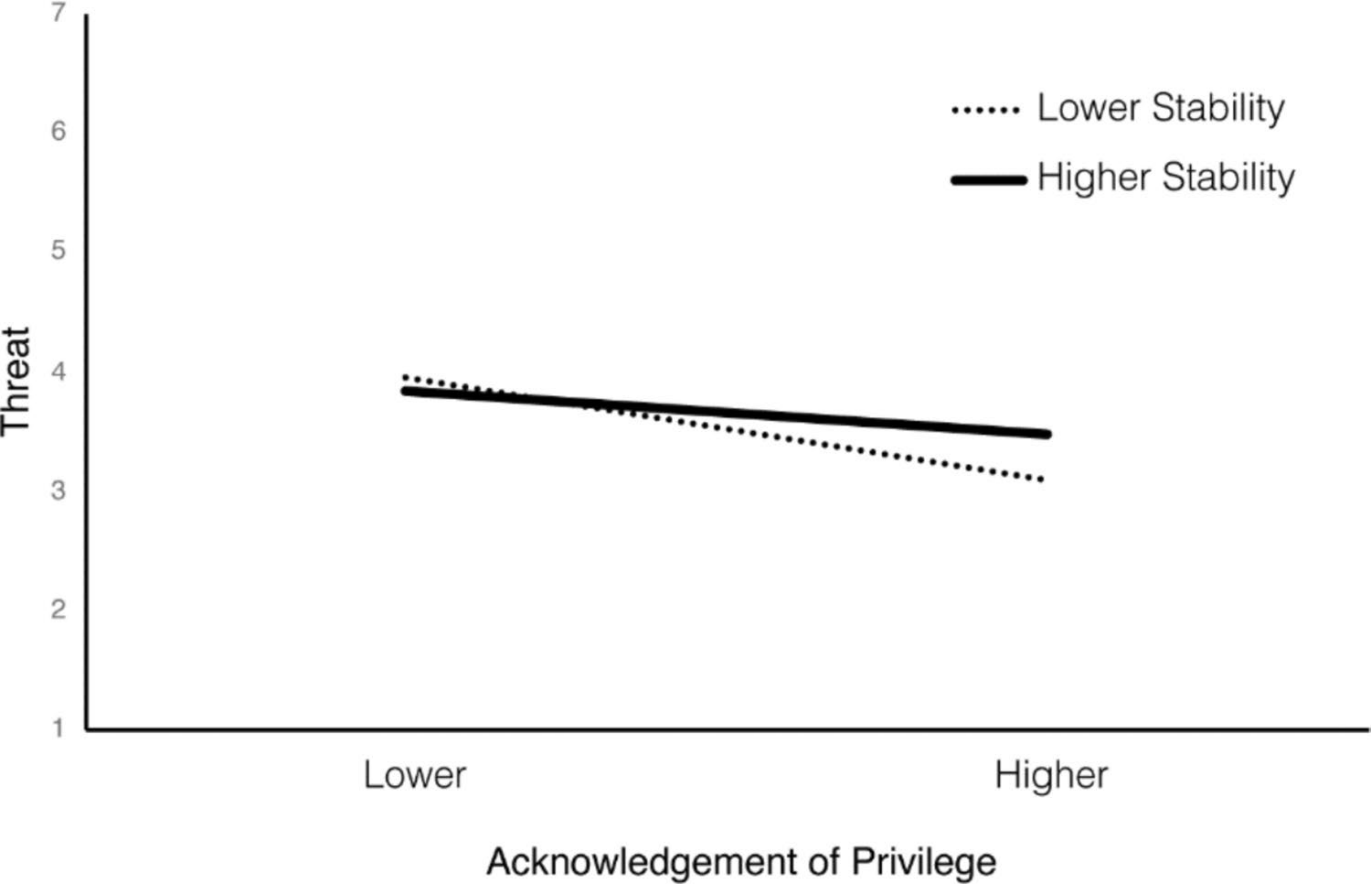
Influence of privilege acknowledgement on threat moderated by status stability in Study 2

To examine whether perceived status stability and social identification moderated this mediation (Hypotheses 4b and 4c), we calculated indices of moderated mediation (Hayes, 2015) and summarized these in Table 4. We then investigated the nature of significant effects at higher (+ 1 *SD*) and lower values (− 1 *SD*) of the moderator (conditional indirect effects; CIE). In Study 2, we found that the indirect effect of privilege acknowledgement on manosphere attitudes was conditional on status stability. However, the nature of this effect did not support H4b. The effect of privilege acknowledgement on manosphere attitudes, through threat, was strongest under lower (CIE_anti-fem_ = − 0.16, *p* < 0.001; CIE_evol_ = − 0.10, *p* < 0.001) compared to higher levels of perceived status stability (CIE_anti-fem_ = − 0.07, *p* = 0.010; CIE_evol_ = − 0.04, *p* = 0.021). As visualized in Fig. 4, this was due to the direct relationship between privilege acknowledgement and threat being strongest when participants saw the status quo as relatively unstable (*B* = − 0.33, *p* < 0.001), compared to stable (*B* = − 0.14, *p* = 0.001). As perceived status stability decreased, higher levels of privilege acknowledgement were associated with lower threat. In turn, this reduced threat was associated with lower manosphere attitude endorsement. Finally, we found no evidence to support H4c; the indirect effect of privilege acknowledgement was not conditional on the combined influence of status stability and social identification.

## Discussion

In this research, we examined the role of specific social identity factors related to perceived status privilege, level of social identification, and perceived threat in predicting the endorsement of manosphere attitudes within the broader population of adult male Americans. To achieve this, we considered literature on manosphere ideology (Dickel & Evolvi, 2022; Ging, 2019; Krendel, 2020; Thorburn et al., 2022; Vallerga & Zurbriggen, 2022; Van Valkenburgh, 2021) from a social identity perspective (Branscombe, 1998; Branscombe et al., 1999; Doosje & Ellemers, 1997; Tajfel & Turner, 1986).

We found strong support for our first Hypothesis. The less men acknowledge the gender privileges relative to women, the more likely they are to endorse the manosphere’s narratives towards feminism and evolution. These latter attitudes, particularly how they were currently measured, portray women as innately devious and malicious. This, unfortunately, functions to blame feminism and justify why men are not (to their minds) privileged in society. Moreover, our results indicate that this relationship is partially mediated by men’s experience of feeling threatened by feminism. The mechanism through which lower privilege acknowledgement may influence manosphere attitudes was partially explained by higher levels of perceived threat when interacting with feminists—people who openly assert that men do possess societal status privileges. This finding supported our fourth Hypothesis (H4a), indicating that men who do not acknowledge a privileged status experience more threat, which in turn, is associated with stronger endorsement of manosphere attitudes.

In Study 2, we demonstrated that the perceived stability of social status between men and women moderates the above relationship. Contrary to our predictions, our research shows that men who do not acknowledge their privilege report feeling relatively threatened by feminism and tend to endorse manosphere attitudes, regardless of whether they perceive their status as stable or unstable. On the other hand, men who acknowledge their privileged status and the evolving status dynamic between genders find feminism less threatening and express lower endorsement of manosphere attitudes. We explore what may have driven this unexpected finding in the limitations and future directions section.

Before delving into this, our studies reveal three findings regarding the potential impact of social identification on manosphere attitudes that warrant discussion. First, we found that the relationship between privilege acknowledgement and manosphere attitudes towards feminism depended on two factors: perceived status stability and social identification. For men with lower social identification levels, perceptions of status stability had little relationship with anti-feminist beliefs. Conversely, among men who more strongly identified with their gender, we observed an interaction between privilege acknowledgement and perceived status stability, as described above. Interestingly, we did not find the same pattern of results for manosphere evolutionary beliefs about women’s supposed manipulative nature towards men. A likely reason for this is that attitudes towards feminism and attitudes towards privilege are both closely tied to political ideology, whereas the use of evolutionary concepts and processes as a basis to legitimise beliefs about women’s supposed manipulative nature is not. Hence, we found only partial support for our third hypothesis.

However, our second key finding indicates that the indirect impact of privilege acknowledgement on manosphere attitudes remained consistent across participants, irrespective of their level of social identification. Therefore, we found no support for H4c. Third, the most robust discovery concerning social identification was that men with higher levels of social identification exhibited a greater propensity to feel threatened by feminists and support manosphere attitudes. We will explore this further, along with the topics discussed above, in the section on limitations and future directions below.

### Limitations and Future Directions

To begin, we must discuss several operational limitations of our research. Firstly, inferences on the observed effect of perceived threat are necessarily limited by our chosen measure, which focused on participants’ experiences of threat-related emotions when interacting with feminists. Hence, the operationalisation processes may not hold true among men who avoid all contact with feminists. If those men were in the current sample, we suspect that their response could easily have been indifference (scale midpoint); others, of course, may have been guided by the content of particular stereotypes, in which case they may have answered at one or the other end of the response scale. Although we have no reason to believe that responses of this potential subgroup of men would be anything but random on this measure in our study, it would be valuable for future research to measure intergroup contact (e.g., Lolliot et al., 2015) and examine its potentially moderating process. Secondly, we estimated perceived status stability from two items. Although these two items demonstrated significant internal consistency in both studies, they likely do not adequately cover the full breadth of the concept. Further, these two items used to estimate perceived status stability asked participants whether they could envision changes in the relationships between men and women. While participants might have interpreted these items in terms of the gender status quo (as intended), some may have understood them as referring to the stability of relationships between men and women more broadly. Moreover, perceptions of stability in gender relations might mean very different things to different people, depending on their foundational beliefs regarding gender status. For instance, the same level of reported instability could reflect the perception that gender relationships are unstable because women are gaining status over men or because women are making strides towards gender equality.

The subjective meaning of this potential status instability may also be influenced by an individual's perception of change as either positive or negative, further complicating its interpretation in our research. To address these uncertainties, future research should develop a more explicitly worded measure of gender stability. This measure could capture not only the perception of change but also the specific direction of that change and the valence attributed to such change.

In addition, a critical factor that may have impacted participants' abilities to imagine future change could have been the degree to which they essentialised gender relations (David et al., 2004; Grace et al., 2015). Hence, perceptions of relative status stability may inadvertently capture an aspect of manosphere ideology: the essentialist nature of gender relations. Those who adhere to this may struggle to envision changing gender relations because they believe the traditional status quo is immutable. Conversely, those who do not adhere to essentialised gender relations may more readily anticipate and welcome future social change. Although we did not measure gender essentialism, this interpretation is supported by literature indicating that higher gender essentialism correlates with more favorable views of gender inequality and scepticism regarding the feasibility of social change (Morton et al., 2009; Skewes et al., 2018).

With this in mind, we propose two psychological processes that may explain the unanticipated moderating effect of status stability observed in our research. First, we must consider why acknowledging privilege did not diminish perceived threat and anti-feminist attitudes among men who strongly identify with their gender and cannot envision changes in gender relations. It is plausible that these men, even when acknowledging their privilege, may rationalise the status quo as natural or inevitable, thereby perceiving their privilege as deserved and, hence, opposing any change. This rationale might lead to the perception of feminist actions as extreme or unfair, engendering a sense of threat and leading to the adoption of manosphere attitudes as a way to push back. In contrast, men who identify strongly with their gender but do not hold gender essentialist views might acknowledge the illegitimacy of their privilege, which in turn could foster receptiveness to social change, a positive stance towards feminists, and a rejection of manosphere attitudes. Clearly, these are potential social and psychological processes that warrant further empirical research.

Of course, it is worth noting that although most of our findings were replicated across both studies, the one exception to this was the two-way interaction between privilege acknowledgement and status stability on threat, which emerged only in Study 2. To further contextualise the significance of this finding, we calculated the effect size for this term, resulting in *f*^2^ = 0.02. While modest, this effect size meets the study’s power threshold (*f*^2^ ≥ 0.017) as indicated by our power analysis. This finding suggests that, although small, the interaction effect is likely to be meaningful, not merely due to chance, and also warrants further investigation to assess its robustness across different contexts and samples.

We also recognize that the correlational and cross-sectional nature of our research means that the results do not provide evidence to support causal pathways. Indeed, it is equally valid to argue that our findings may imply the opposite direction of cause-effect. For example, the rejection of manosphere attitudes towards feminism (i.e., a pro-feminist stance) may lead men to feel more comfortable with feminists, and in turn, more likely to acknowledge their privilege. As articulated above, we proposed the opposite pathway due to our theoretical framework of social identity-related factors, the causality of which has been supported by experimental research in other contexts (e.g., Maass et al., 2003; Morton et al., 2009). Nonetheless, it is accurate to say that our selected constructs of privilege acknowledgement, status stability, perceived threats from feminism, and manosphere attitudes towards feminism are interconnected concepts, particularly because of their association with political ideology. Hence, the relationship between these constructs may be reciprocal. For example, feeling threatened by feminists may lead men to deny their privilege (thus framing feminists as illogical and justifying their discomfort), which then strengthens the threat they perceive from feminism.

To delve deeper into these dynamics, we suggest experimental research investigating the effects found in our research. For instance, research could determine whether exposure to evidence advocating a biological underpinning for gender status (e.g., see Morton et al., 2009) amplifies perceived threats and the endorsement of manosphere attitudes in men with strong gender identification, as opposed to those presented with evidence that supports the social construction of gender roles.

Another interesting avenue for future research could be to investigate the meaning behind the robust main effect of men’s social identification we observed. Central to this inquiry is the question of what men's gender identification means and its connection to ideological beliefs. Indeed, Cameron and Lalonde (2001) suggested that social identification stems not solely from one’s gender category but also from attitudes towards sex roles and relations, leading to various subtypes of identification, such as traditional and non-traditional gender identification (see also David et al., 2004). While substantial research has focused on women, notably examining the distinct effects of feminist versus traditionalist identifications on collective action (e.g., Nelson et al., 2008; Van Breen et al., 2017), similar scrutiny has not been extended to men.

To address this, future studies could examine masculine identification through Van Breen et al.’s (2017) multidimensional approach, which distinguishes women’s gender identification into content identification (the meaning of being a woman, e.g., femininity) and politicized identification (the social positioning of being a woman, e.g., relative disadvantage). For example, future studies could investigate whether certain combinations of identification—specifically, strong alignment with both the content and political dimensions (particularly perceived disadvantage)—correlate with contemporary expressions of sexism within the manosphere. Indeed, such a pattern of identification resembles what Ging (2019) termed hybrid masculinity in the manosphere, characterised by the concurrent embrace of traditional masculine identity and perceived victimhood.

Thirdly, our research focused on specific manosphere attitudes towards feminism and evolutionary beliefs about women’s manipulative nature that, while common, do not capture the full spectrum of sexist beliefs expressed in this diverse online community. For example, research has identified that contemporary sexism in the manosphere is also expressed through themes of male entitlement (i.e., the belief that men have an inherent right to sex, power, and privileges; Hopton & Langer, 2022), particularly aggrieved entitlement in incel forums (e.g., believing that they are unjustly denied access to sex that women owe them; Ging, 2019), and the reduction of women’s worth to their sexual market value (e.g., physical attractiveness; Van Valkenburgh, 2021). Given the recent proliferation of manosphere ideology, via online platforms such as TikTok (Solea & Sugiura, 2023), manosphere-related sexism is likely to be an increasingly prevalent expression of sexism in young people. However, for those interested in researching the causes and consequences of sexism in this population, it is important to note that most measures of sexism (for review, see Ryan & Zehnter, 2022) were created and validated during a time when the manosphere did not exist. Therefore, existing measures do not capture the current language, content, and context of sexism emanating from the manosphere and its inevitable spread from virtual spaces. Together, this signifies a need for future research to develop and validate a new measure that accurately reflects the contemporary nuances of manosphere-related sexism.

### Practical and Theoretical Implications

The robust associations between privilege denial, perceived threat from feminism, masculine identity, and manosphere attitudes found in this research underscore the importance of preventive educational programs aimed at fostering positive masculine identity (e.g., see Wilson et al., 2022) and addressing misinformation and underlying assumptions about gender inequality, particularly in school-aged children who are increasingly encountering manosphere narratives online (Reset Australia, 2022). In doing so, such programs can provide young people with the tools to recognize the societal privileges that various groups of people receive simply because of their social category (including gender). Approaching this with an empathic and non-confrontational manner, for example, noting that gender-based privileges do not negate relative disadvantages in other domains (e.g., race, ethnicity, and low SES), encouraging a critical approach to online content, and developing a positive masculine identity would be important.

Our research also contributes to the expanding body of literature examining perceived privilege loss among traditionally high-status groups (Carian, 2022; Hodson et al., 2022; Zehnter et al., 2021), advancing our understanding of the complexities surrounding subjective perceptions of status privilege. This work challenges the theoretical assumption that men uniformly view themselves as part of a high-status group and emphasises the importance of investigating subjective perceptions of status privilege.

Additionally, our findings on the role of stability in gender relations, consistent across two studies, suggest a need to reassess how we conceptualise the role of instability in contemporary gender discourse. In contrast to our predictions derived from social identity theory (Branscombe et al., 1999; Tajfel & Turner, 1986), the current results suggest that envisaging future instability can, under specific circumstances, attenuate threat and prejudice. These circumstances are likely to pertain specifically to the perceived illegitimacy of male privilege, an additional social identity theory variable worthy of further investigation (e.g., Brandt et al., 2020). These insights necessitate a reorientation in future theoretical models that prioritize factors that shape perceptions of gender-related societal shifts (e.g., gender essentialism) and their interplay with emerging forms of sexism.

## Conclusion

Our research takes a step toward understanding manosphere attitudes’ social and psychological underpinnings. As anticipated, we identified that men who do not acknowledge relative privilege are more likely to feel threatened by feminism, which in turn, is associated with higher endorsement of manosphere attitudes. However, we found that simply acknowledging one’s privilege is not enough to reduce manosphere attitudes among men who strongly identify with their gender and believe that social change is unlikely. We suggest this finding may be driven by underlying gender essentialism, which perpetuates the belief that men deserve their privilege and that progress towards gender equality is unfeasible. We encourage future research to test the role of gender essentialism, expand our understanding of the meaning of men’s social identification, and develop and validate a new measure of manosphere-related sexism. By understanding the factors that lead men to endorse sexist attitudes in the manosphere, as well as the content and prevalence of these attitudes, we can better inform future research and begin to understand how to effectively intervene and empower young people to resist the harmful ideologies of the manosphere.

## Data Availability

The data and code are available at https://osf.io/2wek6/?view_only=0a83235d35db48eab9e29e555470a262.

## Footnotes

1 Where manosphere forums and blogs are referred to, these are articulated using the forum or blog name (e.g., “rational male blog”). All other citations refer to studies analysing manosphere ideology.

2 We note a comprehensive review of the use and misuse of evolutionary psychology in the manosphere is beyond the scope of this paper.

3 The wording of the hypotheses presented in this manuscript slightly differs from those stated in our pre-registration. This modification was made to enhance conceptual clarity and improve communication. However, it is important to note that these changes do not alter the fundamental predictions of our study. The core hypotheses remain consistent with our initial theoretical framework and research intentions as outlined in the pre-registration (https://aspredicted.org/TTP_MZJ).

4 The surveys included additional exploratory measures not reported in this paper.
